# A Rapid and Semi-Quantitative Gold Nanoparticles Based Strip Sensor for Polymyxin B Sulfate Residues

**DOI:** 10.3390/nano8030144

**Published:** 2018-03-05

**Authors:** Yue Li, Liqiang Liu, Shanshan Song, Hua Kuang, Chuanlai Xu

**Affiliations:** 1 State Key Laboratory of Food Science and Technology, Jiangnan University, Wuxi 214122, China; liyuejiangnan@163.com (Y.L.); murel@163.com (L.L.); sss@jiangnan.edu.cn (S.S.); 2 International Joint Research Laboratory for Biointerface and Biodetection, and School of Food Science and Technology, Jiangnan University, Wuxi 214122, China; 3 Collaborative Innovationcenter of Food Safety and Quality Control in Jiangsu Province, Jiangnan University, Wuxi 214122, China

**Keywords:** polymyxin B, immunochromatographic test strip, gold nanoparticles, monoclonal antibody, milk, animal feed

## Abstract

Increasing attention is now being directed to the utilization of polymyxin B (PMB) as a last-line treatment for life-threatening infections caused by multidrug resistant Gram-negative bacteria. Unfortunately, polymyxins resistance is also increasingly reported, leaving a serious threat to human health. Therefore, the establishment of rapid detection methods for PMB residues is highly essential to ensure public health. In this study, two monoclonal antibodies (mAb; 2A2 and 3C6) were obtained using PMB-bovine serum albumin as the immunogen and PMB-ovalbumin as the coating antigen, which were prepared with *N*-(γ-maleimidobutyryloxy) succinimide ester and glutaraldehyde as cross-linking agents, respectively. Through an indirect competitive enzyme-linked immunosorbent assay, resultant two mAbs were compared and the results indicated that 3C6 showed higher sensitivity with a half maximum inhibition concentration of 13.13 ng/mL. Based on 3C6, a gold nanoparticles (AuNPs)-based immunochromatographic test (ICT) strip was then established, the mechanism of which is that free PMB competes with the fixed coating antigen to combine with mAb labeled by AuNPs. Using ICT strip to detect milk and animal feed samples revealed the visible detection limits were 25 ng/mL and 500 μg/kg, respectively and the cutoff limits were 100 ng/mL and 1000 μg/kg, respectively. The ICT strip provides results within 15 min, facilitating rapid and semi-quantitative analysis of PMB residues in milk and animal feed.

## 1. Introduction

**Figure 1:**
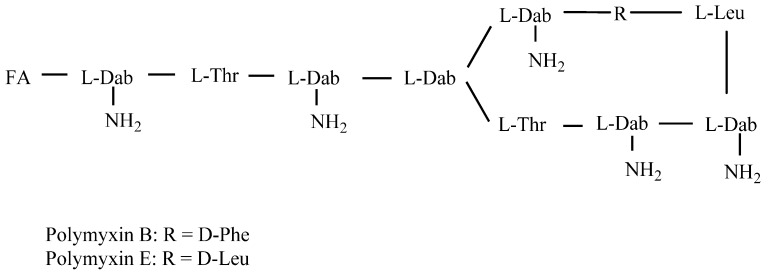
Chemical structures of polymyxin B and E.

Polymyxin B (PMB), belonging to the family of polymyxins, is a lipopeptide antibiotic isolated from *Paenibacillus polymyxa* [1,2]. Structurally, it consists of a cyclic heptapeptide tripeptide side chain and a fatty acid tail (Figure 1) [3]. Although PMB possesses broad spectrum activity against many species of Gram negative bacteria by binding to the lipid A moiety of the bacterial lipopolysaccharide and subsequently disintegrating the bacterial membranes [4,5,6], it was unfavorable in the 1960s due to its toxic side effects, such as nephrotoxicity and neurotoxicity [7,8]. However, owing to the current inexorable emergence of multidrug resistant (MDR) Gram negative bacteria and the decline in newly developed antibiotics, PMB has been increasingly used clinically as a last resort drug for the treatment of MDR Gram negative bacterial infections [9,10,11]. 

Nevertheless, with the marked increase in usage of polymyxins to treat humans and animals, bacterial resistance has also increasingly emerged, leaving a global challenge in terms of polymyxins utilization [12,13,14]. Except for being utilized in the clinic, PMB is also used as a feed additive in animal husbandry to promote growth. However, at present, there is no maximum residue limit for PMB in animal-derived food products or animal feed nationally or internationally. Consequently, the abuse of PMB in animal food production has increased, resulting in a massive accumulation of PMB in animal-derived food products, which may put animals and ultimately, humans at risk for the acquisition of antimicrobial drug resistant pathogens [15]. Thus, to prevent this situation, the quantification of PMB in food and animal feed should be a focus of attention in terms of surveillance screening.

PMB and polymyxin E (PME) only differ in a single amino acid residue within the heptapeptide ring (Figure 1) and are both used in clinical practice and animal production [3]. Commercially, PMB is available as a sulfate salt, a mixture of over 30 polypeptide species with the major components consisting of polymyxin B1, B1-1, isoleucine-polymyxin B1 (Ile-PMB1), B2, B3, B4, B5 and B6, differing in their fatty acid moiety [16]. PME is used as a sulfate salt or colistimethate sodium [2].

The most commonly used method for the quantification of PMB is currently chromatography. Several liquid chromatography tandem mass spectrometry methods have been developed for the quantification of: (i) polymyxin B1 and B2 in cation-adjusted Mueller-Hinton broth, human and rat plasma using protein precipitation extraction (PPE) for sample preparation [17]; (ii) polymyxin B1, B2 and B1-1 in human plasma and treated human urine utilizing solid phase extraction (SPE) for sample preparation [18]; (iii) polymyxin B1, B2, B3 and isoleucine-polymyxin B1 in human plasma, using trichloroacetic acid (TCA) and TCA-facilitated PPE [19]; and (iv) polymyxin B in bacterial growth media using PPE [20]. However, despite its sensitivity and accuracy, chromatography requires complex sample preparation, numerous organic solvents and expensive instruments, which restricts its wide-scale application. As an alternative, immunoassay is sensitive, simple and eco-friendly, as it is time saving, economical and does not require organic solvents. An enzyme-linked immunosorbent assay (ELISA) was established for the detection of PMB sulfate in human serum [21]. However, this assay only detected PMB sulfate in human serum samples and was not suitable for on-site analysis of PMB as a period of more than 3 h is required. Consequently, the development of a sensitive, rapid and reliable method is imperative for detection of PMB residues in animal-derived food products and animal feed samples. 

Recently, the groundbreaking developments of nanoparticle-based biosensors offer many technological advances in food safety monitoring [22]. Nanoparticles are used commonly as labels for signal transduction and amplification [23,24]. Amongst nanoparticles, gold nanoparticles (AuNPs) are used most widely due to its strong adsorption and stability, which makes them suitable for ultrasensitive detection [25]. Yet, to our knowledge, no rapid and AuNPs-based analysis method for PMB has been reported for animal-derived food products and animal feeds. Therefore, in the present study, we prepared mouse-derived monoclonal antibodies against PMB with high sensitivity and specificity and established an AuNPs based immunochromatographic test (ICT) strip for the rapid and semi-quantitative analysis of PMB residues in milk and feed additive samples. 

## 2. Experimental Section

### 2.1. Reagents and Materials

PMB and PME were purchased from J&K Scientific Ltd. (Beijing, China). Bovine serum albumin (BSA), ovalbumin (OVA), *N*-[γ-maleimidobutyryloxy] succinimide ester (GMBS), glutaraldehyde (GA), 3,3′,5,5′-tetramethylbenzidine (TMB) and complete and incomplete Freund’s adjuvants were obtained from Sigma-Aldrich (St. Louis, MO, USA). Horseradish peroxidase (HRP)-labeled goat anti-mouse immunoglobulin (IgG) was obtained from Jackson Immuno Research Laboratories (West Grove, PA, USA). Cell culture media were supplied by Life Technologies Corporation (Shanghai, China). Animal feed was purchased from Qinglongshan Laboratory Animal Co., Ltd. (Nanjing, China). All other reagents were purchased from the National Pharmaceutical Group Chemical Reagent Co., Ltd. (Shanghai, China).

Polyvinylchloride (PVC) backing card, glass fiber membrane, nitrocellulose (NC) membrane, sample pad (CB-SB08) and absorbent pad (SX18) were purchased from JieYi Biotechnology Co., Ltd. (Shanghai, China). The Dispensing Platform was obtained from Kinbio Tech Co., Ltd. (Shanghai, China). 

All buffer solutions were prepared with ultrapure water (Milli-Q purification system, Millipore Co., Bedford, MA, USA).

### 2.2. Synthesis of PMB-Protein Conjugates

**Figure 2:**
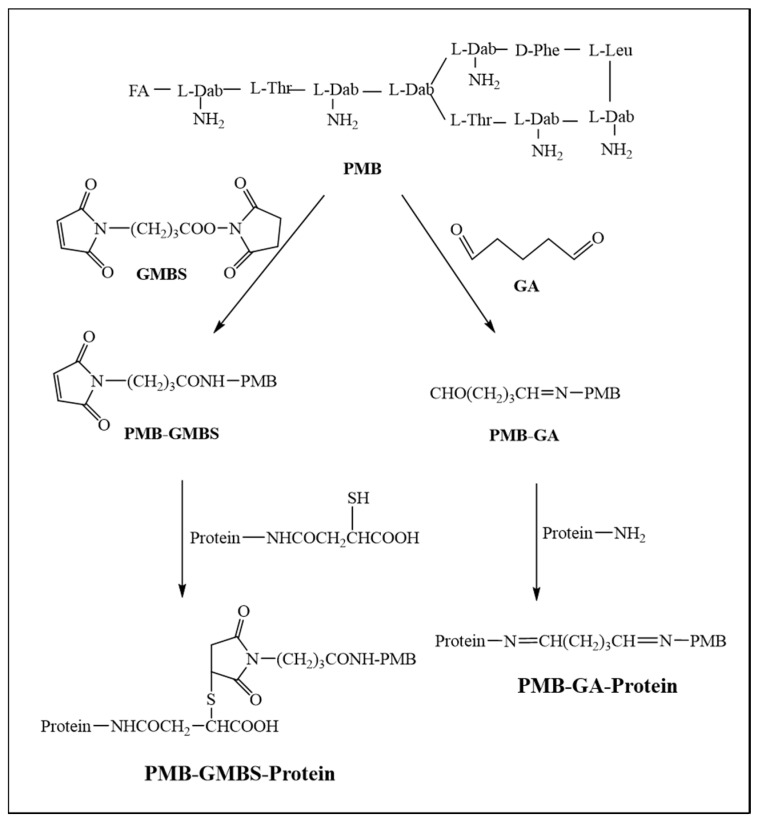
Conjugation of PMB to carrier proteins.

PMB was coupled to BSA with GMBS as a cross-linker according to a modified procedure previously described (Figure 2) [21,26]. In brief, 14 mg of PMB in 1.5 mL 0.1 M phosphate-buffered saline (PBS) (pH = 7.4) was added to GMBS (2.8 mg), dissolved in 0.5 mL tetrahydrofuran and stirred for 30 min at 30 °C. Following nitrogen gas treatment, the resulting mixture was washed three times using ether:methylene chloride (2:1, *v*/*v*) to remove excess GMBS to obtain solution A. Ten mg of BSA was dissolved in 0.2 mL 0.1 M PBS and added to 50 µL 0.5 M hydroxylamine. After stirring for 10 min at 25 °C, the mixture was diluted with 3 mL of 0.1 M PBS (containing 3 M urea) to obtain solution B. Subsequently, solution B was immediately added to solution A and vigorously stirred at room temperature for 30 min. The resultant mixture was dialyzed against 0.01 M PBS for 3 days. The resulting solution was utilized as the immunogen and defined “PMB-GMBS-BSA.” Similarly, the conjugate of PMB and OVA, used as the coating antigen, was prepared using the same method and defined “PMB-GMBS-OVA.” 

Another coating antigen was synthesized with GA as the cross-linker (Figure 2) [27,28] and labeled “PMB-GA-OVA1.” Briefly, 14 mg of PMB and 48 µL of 2.5% GA were subsequently dissolved in 200 µL of ultrapure water. After mixing at room temperature for 2 h, the activated solution was added dropwise into 1 mL of a stirred solution of 5 mg of OVA in 0.01 M PBS (pH = 7.4) and stirred for 4 h at room temperature. Following the same procedure, PMB-GA-OVA2 was prepared by changing 0.01 M PBS to 0.01 M MES (pH = 5.6) to dilute OVA. The resultant mixtures were dialyzed as above and then stored at −20 °C. 

### 2.3. Production and Purification of mAbs

The mAbs were produced using a previously described procedure with slight modifications [29]. Female BALB/c mice (8–10 weeks of age, Qinglongshan Laboratory Animal Co., Ltd. (Nanjing, China)) were immunized with an emulsion prepared from 100 μg immunogen in 100 μL normal saline with an equal volume of complete Freund’s adjuvant by subcutaneous multipoint injection. Four weeks after the initial injection, four booster injections were given triweekly with 50 μg immunogen mixed with an equal volume of incomplete Freund’s adjuvant. Finally, the mice with highest affinity and lowest IC_50_ to PB were given an intraperitoneal injection of 25 μg immunogen in 100 μL normal saline. Three days after the final injection, spleen cells were collected and fused with SP2/0 via PEG 1500 according the same procedure [23]. After three subclones, two hybridomas were raised and expanded in BALB/c mice. Finally, the mAbs were purified using the caprylic acid-ammonium sulfate method [30].

### 2.4. IcELISA Procedure

The icELISA was performed according to conventional protocols [31]. In brief, 100 μL of coating antigen diluted in coating buffer (0.01 M carbonate buffer, CBS, pH 9.6) was coated in 96-well microplates. After incubating at 37 °C for 2 h, the plates were washed three times with washing buffer (0.05% (*v*/*v*) Tween-20 in 0.01 M PBS) and each well was subsequently blocked with 200 μL of blocking buffer (0.05 M CBS containing 0.2% (*m*/*v*) gelatin) for 2 h at 37 °C. Following another washing step, 50 μL of a serially diluted PMB standard solution or sample solution was added to different wells, then 50 μL of anti-PMB mAb was added to each well and the plates were incubated at 37 °C for 30 min. Similarly, 100 μL of HRP-IgG was added to each well and incubated for 30 min at 37 °C after a wash procedure. After washing four times, 100 μL of TMB substrate was added to each well to react for 15 min at 37 °C in darkness. Subsequently, the enzymatic reaction was stopped by the addition of 50 μL of 2 M sulfuric acid per well. The absorbances were measured at 450 nm with a microplate reader.

### 2.5. Specificity of mAbs

The antibody specificities were determined by measuring cross-reactivity (CR) with PME using the icELISA method. The CR values were calculated according to the following equation:

CR% = (IC_50_ of PMB)/(IC_50_ of PME) × 100

### 2.6. Preparation of AuNPs-Labeled mAb

The AuNPs solution was fabricated using sodium citrate reduction method as previously described [32], with some modifications. In brief, 100 mL of 0.01% (*w*/*v*) chloroauric acid solution was boiled under vigorous stirring and then mixed with 2.0 mL of freshly prepared 1% (*w*/*v*) trisodium citrate solution. The mixture solution was stirred unceasingly until the color turned to wine-red. After boiling for a further 5 min, the solution was cooled to room temperature and stored at 4 °C until used.

The antibody was labeled with AuNPs based on a previously described protocol [33]. Briefly, 10 mL of 1 nM AuNPs solution was adjusted to pH 8.0 with 0.1 M potassium carbonate solution and then conjugated with the anti-PMB mAb. Following incubation at room temperature for 1 h, the conjugates were mixed with 1 mL of 0.5% (*w*/*v*) BSA at room temperature for another 2 h to stabilize the conjugates. Subsequently, the mixture was centrifuged at 7000× *g* for 30 min. Finally, the resulting precipitate was washed three times with 0.02 M phosphate buffer (containing 5% sucrose, 1% BSA and 0.5% PEG-6000, pH 7.4), resuspended in 1 mL of 0.02 M PBS containing 0.02% NaN_3_ and stored at 4 °C until use.

### 2.7. Construction and Principle of the Immunochromatographic Test Strip

**Figure 3:**
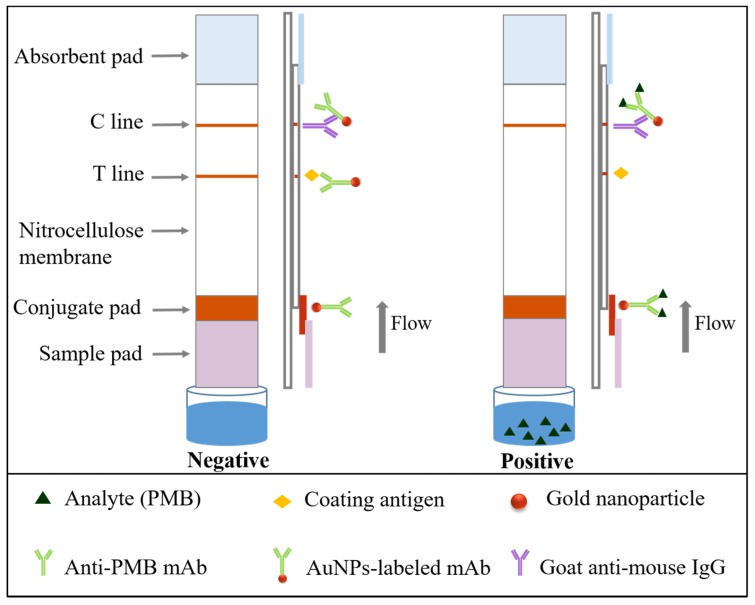
The scheme and principle of gold nanoparticles based immunochromatographic strip.

As shown in Figure 3, the composition of the 5 cm ICT strip, starting from the bottom, was as follows: the sample pad, the conjugate pad, the NC membrane and the absorbent pad [34]. The conjugate pad was sprayed with AuNPs-labeled mAb and two types of mAb were used. For the test line (T line) and control line (C line), coating antigen (two types of coating antigen were tested) and goat anti-mouse IgG were respectively sprayed on the NC membrane. The sample pad and absorbent pad were stuck to each end of the PVC backing card. All membranes were dried for 30 min at 37 °C. 

In the present study, the ICT strip is performed based on a competitive reaction between the free PMB contained in the sample and the fixed coating antigen applied onto the NC membrane to combine with AuNPs-labeled mAb. Owing to capillary action, sample solution added to the sample pad migrates towards the absorption pad. If the sample is analyte-positive, AuNPs-labeled mAb binds to free PMB first during diffusion, resulting in lower AuNPs-labeled mAb triggered by the coating antigen fixed on the T line, ultimately leading to a lighter T line. As the concentration of PMB increasing, the color of the T line disappeared. On the contrary, if the sample is analyte-negative, AuNPs-labeled mAb only bound to coating antigen and goat anti-mouse IgG, resulting in a visible T line and C line. The C line should always emerge, indicating the effective assembly of the strip. 

### 2.8. Sample Analysis

Milk and animal feed samples were analyzed using the ICT strips. All samples were confirmed as PMB negative by LC-MS/MS. The samples were then spiked with various amounts of PMB reference and the feed samples were pretreated for analysis as follows: In brief, a representative sample was first ground and mixed. Then 5.0 g of the ground sample was added to 25.0 mL of 70% methanol and vortexed vigorously for 3 min. Following centrifugation for 5 min at 4000× *g* at room temperature, 1 mL of the obtained supernatant was diluted with 1 mL of distilled water and analyzed by the ICT strip. 

## 3. Results and Discussion

### 3.1. Identification of Conjugates

**Figure 4:**
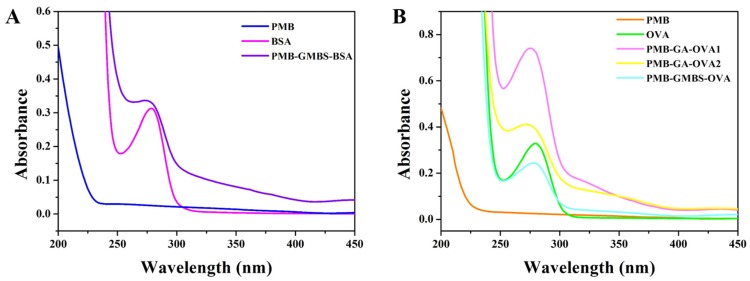
UV-vis spectra of BSA (0.5 mg/mL), PMB (0.5 mg/mL) and PMB-GMBS-BSA (0.5 mg/mL) (**A**) and (**B**) OVA (0.5 mg/mL), PMB (0.5 mg/mL) and the conjugates (PMB-GA-0VA1, PMB-GA-OVA2 and PMB-GMBS-OVA) (0.5 mg/mL).

Confirmation of the conjugation reactions was accomplished using ultraviolet–visible (UV–vis) spectra in the range of 200–450 nm (Figure 4). There was no characteristic absorbance contribution of PMB, while the two carrier proteins, BSA and OVA, showed characteristic absorbance at 280 nm. The characteristic absorbance peaks of the conjugates (PMB-GMBS-BSA, PMB-GMBS-OVA, PMB-GA-OVA1 and PMB-GA-OVA2) reached almost 375 nm. In addition, the wider peaks at 280 nm were observed for all the PMB conjugates. These differences in waveforms and peak type proved that the couplings of PMB and carrier proteins were successful.

### 3.2. Characterization of mAbs

To select the high sensitivity antibody, three types of coating antigens, PMB-GMBS-OVA, PMB-GA-OVA1 and PMB-GA-OVA2, were tested. The inhibition rate (1 − A/A_0_)* 100% was chosen to authenticate the binding ability of antibodies to PMB, where A and A_0_ were the OD_450nm_ values of the PMB standard solution and blank solution, respectively. 

**Figure 5:**
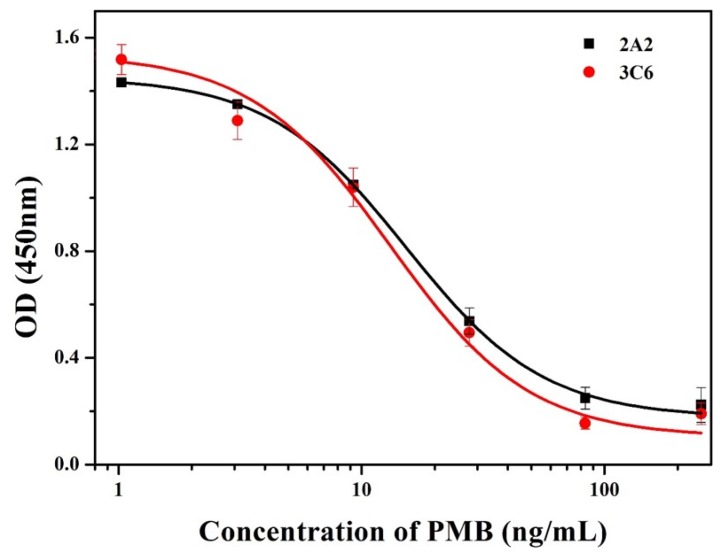
Calibration curves of mAb 2A2 and 3C6.

The data indicated (not shown) that 2A2 showed low inhibition rates, irrespective of the coating antigen used. For 3C6, although homologous coating antigen (PMB-GMBS-OVA) provided higher affinity, the heterologous combination (PMB-GA-OVA1) was chosen for further investigation due to its superior inhibition compared with PMB-GA-OVA2. This may have been due to exposure of the recognition site to heterologous coating antigen [35]. The calibration curves of 2A2 and 3C6 were then established (R^2^ = 0.999, Figure 5). It was found that the IC_50_ values of the two mAbs were 15.26 ng/mL and 13.13 ng/mL, respectively, which revealed that 3C6 showed higher sensitivity. 

### 3.3. Specificity of mAbs

By determining the CR for PME, the specificities of 2A2 and 3C6 were assessed (Table 1). The data showed that both 2A2 and 3C6 exhibited low CR for PME, where the CR values were 5.06% for 3C6 and no CR was obtained for 2A2, indicating that both 2A2 and 3C6 possessed high specificity for PMB. 

### 3.4. Preparation of AuNPs

**Figure 6:**
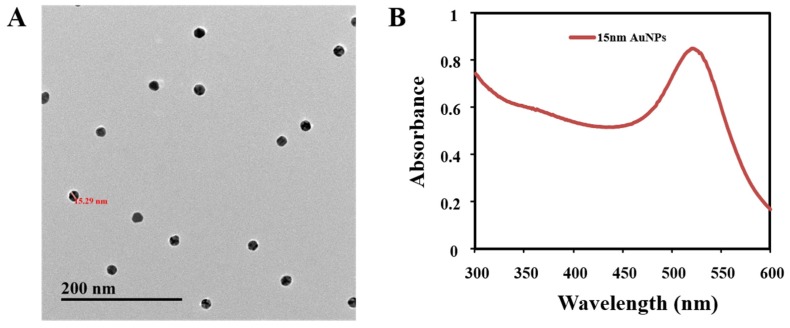
Characterization of the AuNPs solution: (**A**) transmission electron microscopy (TEM) images and (**B**) ultraviolet–visible (UV–vis) spectra.

Through electrostatic bonds, gold nanoparticles can combine with many proteins and retain their bioactivity. In present study, AuNPs with a diameter of 15 nm were selected because of their stability and adsorption [33,36]. As shown in Figure 6, the AuNPs possess predominant homogeneity and maximum absorption at 523 nm. 

### 3.5. Optimization of the ICT Strip

**Figure 7:**
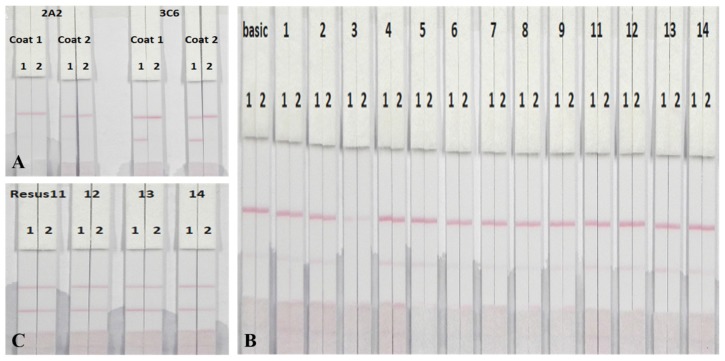
(**A**) Two kinds of AuNPs-labeled mAb with two kinds of coating antigens, respectively. Coating 1 was PMB-GA-OVA1, while coating 2 was PMB-GA-OVA2. The concentration of each coating antigen was 0.5 mg/mL. 1 = 0 ng/mL and 2 = 250 ng/mL of PMB standards. (**B**) ICT strip with different running buffers. The basic buffer contained 20 Mm Tris buffer (pH 8.2), 0.1% (*w*/*v*) PEG, 0.1% (*w*/*v*) Tween-20, 5% (*w*/*v*) sucrose, 5% (*w*/*v*) thehalose and 0.2% (*w*/*v*) BSA. Other buffers, from left to right, including PVP, PEG, PVA, BSA, casein, sucrose, thehalose, sorbitol, mannitol, tween-20, brij 35, triton X-100 and On-870. Coating (PMB-GA-OVA1) concentration was 0.25 mg/mL. 1 = 0 ppb and 2 = 200 ppb. (**C**) Different running buffer with coating (PMB-GA-OVA1) concentration of 0.5 mg/mL, from left to right: tween-20, brij 35, triton X-100 and On-870. 1 = 0 ng/mL and 2 = 250 ng/mL of PMB standards. *n* = 6.

The sensitivity of the ICT strip largely depended on the AuNPs-labeled mAb and the coating antigen immobilized on the conjugate pad and NC membrane, respectively [37]. Therefore, two types of mAb (2A2 and 3C6) and coating antigens (PMB-GA-OVA1 and PMB-GA-OVA2) were compared using 0 ng/mL and 250 ng/mL of the PMB standard solution. As shown in Figure 7A, no test line was observed when mAb 2A2 was combined with PMB-GA-OVA1 or PMB-GA-OVA2, whereas a distinct test line was obtained when 3C6 was combined with both coating antigens. This may be explained by the lower affinity of 2A2 to both coating antigens, as an overt control line was ultimately observed. In addition, for 3C6, an appreciably deeper C line using PMB-GA-OVA1 was observed, which was more beneficial for visual semi-quantitative detection. Consequently, PMB-GA-OVA1 and 3C6 were chosen as the optimal combination for further experiments.

Running buffer has an influence on the flow rate of the AuNPs-labeled mAb and therefore, the intensity of the control line [37,38]. As a consequence, different running buffers were prepared and compared by detecting PMB-negative and PMB-positive samples. The concentration of coating antigen was 0.25 mg/mL. The basic buffer contained 20 mM Tris buffer (pH 8.2), 0.1% (*w*/*v*) PEG, 0.1% (*w*/*v*) Tween-20, 5% (*w*/*v*) sucrose, 5% (*w*/*v*) trehalose and 0.2% (*w*/*v*) BSA. Other buffers included polyvinylpyrrolidone (PVP), PEG, polyvinyl alcohol (PVA), BSA, casein, sucrose, trehalose, sorbitol, mannitol, Tween-20, Brij 35, Triton X-100 and Rhodasurf^®^ On-870 (an ethoxylated oleyl alcohol). With the exception of PVA, all buffers gave a colored control line but higher intensities were observed when Tween-20, Brij 35, Triton X-100 and On-870 were used (Figure 7B). Therefore, these four buffers were used to further optimize experiments with 0.5 mg/mL of coating antigen (Figure 7C) [36]. Triton X-100 contributed to a deeper T line and C line, probably due to its pH and ionic strength. Therefore, Triton X-100 was selected as the running buffer.

### 3.6. Sensitivity of the ICT Strip

**Figure 8:**
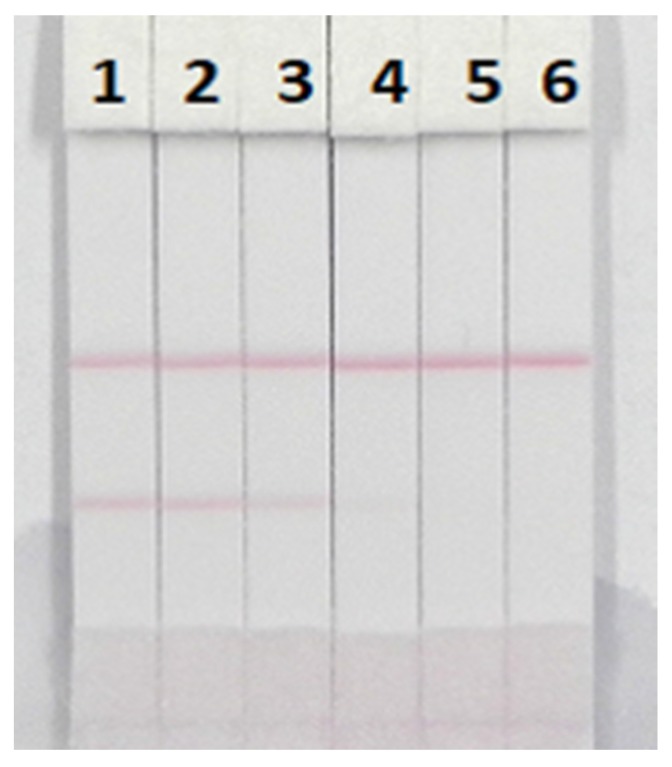
The sensitivity of ICT strip. 1 = 0 ng/mL; 2 = 10 ng/mL, 3 = 25 ng/mL, 4 = 50 ng/mL, 5 = 100 ng/mL and 6 = 250 ng/mL, *n* = 6.

The visual limit of detection (vLOD) of the ICT strip was defined as the lowest concentration of PMB that resulted in a less bright T line compared with that of the negative samples, while the cut-off limit was defined as the threshold concentration of PMB that resulted in complete disappearance of the T line. Under the selected conditions, a series of concentrations of PMB reference substances in ultrapure water were analyzed using the ICT strip. As shown in Figure 8, when the concentration of PMB increased, a lighter T line was observed. Twenty-five ng/mL of PMB generated a distinct visible difference in the color intensity of the T line between the sample and the blank (no PMB). Consequently, 25 ng/mL of PMB was regarded as the vLOD value. In addition, the color of the T line entirely disappeared at 100 ng/mL, which revealed that 100 ng/mL of PMB was the cutoff limit. The sensitivity of the ICT strip was ideal.

### 3.7. Sample Analysis

**Figure 9:**
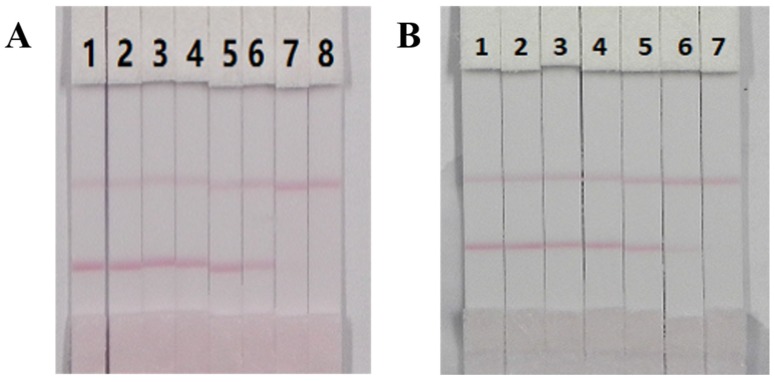
Analysis of samples with PMB spiking using ICT strip. Milk samples (**A**), 1 = 0 ng/mL, 2 = 1 ng/mL, 3 = 2.5 ng/mL, 4 = 5 ng/mL, 5 = 10 ng/mL, 6 = 25 ng/mL, 7 = 50 ng/mL and 8 = 100 ng/mL. Animal feed samples (**B**), 1 = 0 μg/kg, 2 = 25 μg/kg, 3 = 50 μg/kg, 4 = 100 μg/kg, 5 = 250 μg/kg, 6 = 500 μg/kg and 7 = 1 μg/kg. *n* = 6.

The performance characteristics of the ICT strip were determined by analysis of milk and animal feed samples, which were verified as PMB negative by LC-MS/MS (data not shown) and spiked with different amounts of PMB reference substances. The milk samples were spiked with PMB standard solution (10 µg/mL, diluted in 0.01 M PBS, pH 7.4) at final concentrations of 0, 1, 2.5, 5, 10, 25, 50 and 100 ng/mL, while animal feed samples were spiked with PMB at concentrations of 0, 25, 50, 100, 250, 500 and 1000 μg/kg. The spiked series was prepared on different days. In general, an ICT strip approach should be easily conducted within a limited time. Therefore, no sample preparation and a simple sample preparation procedure were required for the milk samples and animal feed samples, respectively. Each sample was analyzed six times with the optimal ICT strips. The results are presented in Figure 9 and show that the color intensity of the T line for the milk samples and animal feed samples weakened when the concentration of PMB increased until the cutoff limits were reached. Significantly weaker T lines compared with the negative samples were observed at 25 ng/mL for the milk samples and 500 μg/kg for the animal feed samples. Furthermore, 100 ng/mL and 1000 μg/kg of PMB were the cutoff limits for the milk samples and animal samples, respectively. Therefore, the ICT strip could be applied for the semi-quantitative detection of PMB in milk and animal feed samples.

## 4. Conclusions

Through mouse immune and cell fusion, two anti-PMB antibodies (2A2 and 3C6) were obtained, of which 3C6 showed high sensitivity (IC_50_ value of 13.13 ng/mL) with the heterogeneous combination of PMB-GMBS-BSA with PMB-GA-OVA1. Furthermore, 3C6 had high specificity for PMB with a CR value against PME of 5.06%. Based on mAb 3C6, a sensitive AuNPs-based immunochromatographic strip was established for the rapid and semi-quantitative determination of PMB in milk and animal feed samples, where the vLOD values were 25 ng/mL and 500 μg/kg, respectively and the cutoff limits were 100 ng/mL and 1000 μg/kg, respectively. The proposed ICT strip can be used for rapid screening and semi-quantitative detection of PMB. 

## Figures and Tables

**Table 1:** The CR values of mAb 2A2 and 3C6 against PME by the ic-ELISA method.

mAb	Analytes	IC_50_ (ng/mL)	CR (%)
2A2	PMB	15.26	100%
	PME	>1000	-
3C6	PMB	13.13	100%
	PME	259	5.07%
